# Prognostic Value of Proadrenomedulin in Severe Sepsis and Septic Shock Is Independent of Etiology and Focus of Infection

**DOI:** 10.1186/2197-425X-3-S1-A518

**Published:** 2015-10-01

**Authors:** R Cicuendez, L Nogales, A Bueno, S Gonzalez De Zarate, D Calvo, C Andres, P Bueno, E Zarca, MF Muñoz, J Bermejo, JM Eiros, F Gandia, D Andaluz-Ojeda

**Affiliations:** Hospital Clínico Universitario Valladolid, Intensive Medicine, Valladolid, Spain

## Introduction

Proadrenomedulin (proADM) is a novel prognostic biomarker employed in pneumonia and more recently in severe sepsis and septic shock. Release kinetics of proADM in this disease has not been studied. in this research we analyze the variation in plasma levels of this prohormone based upon the type of causative agent, the site of infection and the presence of renal failure.

## Methods

Plasmatic levels of ProADM were measured in 110 consecutive patients admitted to the ICU with diagnosis of severe sepsis or septic shock at admission. Clinical and demographic data including age, gender, comorbidities, APACHE II and SOFA scores were collected. Recruited period over 12 months. Statistical analysis: χ^2^ test for categorical variables; comparison of proADM levels based upon etiologic agent, site of infection and presence of renal failure were performed by using repeated measures analysis of variance (ANOVA). Statistical significance: p < 0.05.

## Results

110 patients were recruited; Male 63 %, APACHE II score: 21; SOFA score: 8.5; Septic shock: 86%; ICU mortality: 32.7%; Respiratory focus: 49%; Urological focus: 22.7%; Abdominal focus: 15%; Gram negatives: 33%; Gram positives: 30%; Virus: 13%; Fungi: 5.4%. As was expected, non survivors showed significant higher plasma values of proADM than survivors. the patients with pneumonia as focus of sepsis and viruses as causative agent, showed lower levels of plasma proADM than patients with another focus and/or etiologic agent. in contrast, patients with some degree of acute renal failure showed higher levels of proADM.

See tables: plasmatic levels of proADM at admission (mmol/L; median ± IQR). Statistical significance between columns (p < 0.05) is shown as (*).

## Conclusions

Prognostic value of proADM is preserved independent of etiology, focus of infection and presence of renal failure in severe sepsis and septic shock. When compared, severe sepsis from respiratory tract showed lower values of plasma proADM than other origins of sepsis. Alike, sepsis caused by virus showed lower elevation of plasmatic levels of this molecule. Finally presence of acute renal failure is associated with higher plasmatic values of proADM in both survivors and non-surviving septic patients. Larger researches to confirm these findings are necessary.

**Table 1:** *proADM according etiology and survival.*

	Gram -	Gram +	Virus	Fungi
Survivor	4.9 ± 3.9	3.9 ± 4.2	1.34 ± 0.8 *	3.0 ± 3.8
Non survivors	9.9 ± 5.4	8.5 ± 6	1.0 ± 0.3 *	5.8 ± 1.7

**Table 2:** *proADM according focus and survival.*

	Pneumonia	Urologic	Abdominal	Other
Survivors	1.1 ± 1.8 *	5.3 ± 3.7	5.5 ± 6.2	6.0 ± 4.2
Non survivors	5.1 ± 5 *	11.7 ± 4.8	10.6 ± 6.7	12.5 ± 0

**Table 3:** *proADM according renal failure and survival.*

	Renal failure	No renal failure
Survivors	4.9 ± 4.2 *	1.02 ± 2.0 *
Non survivors	11.5 ± 5.0 *	3.5 ± 5.2 *

